# Glioblastoma Extracellular Vesicle-Specific Peptides Inhibit EV-Induced Neuronal Cytotoxicity

**DOI:** 10.3390/ijms23137200

**Published:** 2022-06-28

**Authors:** Wenbo Zhou, Julia Craft, Alex Ojemann, Luke Bergen, Arin Graner, Aitana Gonzales, Qianbin He, Timothy Kopper, Marie Smith, Michael W. Graner, Xiaoli Yu

**Affiliations:** Department of Neurosurgery, University of Colorado Anschutz Medical Campus, 12700 E 19th Ave, Aurora, CO 80045, USA; wenbo.zhou@ucdenver.edu (W.Z.); julia.craft@cuanschutz.edu (J.C.); alex.ojemann@cuanschutz.edu (A.O.); luke.bergen@cuanschutz.edu (L.B.); arin.graner@cuanschutz.edu (A.G.); aitana.gonzales@cuanschutz.edu (A.G.); qianbin.he@cuanschutz.edu (Q.H.); timothy.kopper@cuanschutz.edu (T.K.); marie.smith@cuanschutz.edu (M.S.); michael.graner@cuanschutz.edu (M.W.G.)

**Keywords:** glioblastoma, meningioma, plasma, tumor cell line, extracellular vesicles, ELISA, phage-display, peptides, neurons, cytotoxicity

## Abstract

WHO Grade 4 IDH-wild type astrocytoma (GBM) is the deadliest brain tumor with a poor prognosis. Meningioma (MMA) is a more common “benign” central nervous system tumor but with significant recurrence rates. There is an urgent need for brain tumor biomarkers for early diagnosis and effective treatment options. Extracellular vesicles (EVs) are tiny membrane-enclosed vesicles that play essential functions in cell-to-cell communications among tumor cells. We aimed to identify epitopes of brain tumor EVs by phage peptide libraries. EVs from GBM plasma, MMA plasma, or brain tumor cell lines were used to screen phage-displayed random peptide libraries to identify high-affinity peptides. We purified EVs from three GBM plasma pools (23 patients), one MMA pool (10 patients), and four brain tumor cell lines. We identified a total of 21 high-affinity phage peptides (12 unique) specific to brain tumor EVs. The peptides shared high sequence homologies among those selected by the same EVs. Dose–response ELISA demonstrated that phage peptides were specific to brain tumor EVs compared to controls. Peptide affinity purification identified unique brain tumor EV subpopulations. Significantly, GBM EV peptides inhibit brain tumor EV-induced complement-dependent cytotoxicity (necrosis) in neurons. We conclude that phage display technology could identify specific peptides to isolate and characterize tumor EVs.

## 1. Introduction

CNS5 WHO Grade 4 IDH-wild type astrocytoma (abbreviated as GBM) is the most common astrocytoma and the deadliest primary brain tumor in adults [1,2]. Current treatments have improved 2- to 5-year survival rates slightly since 2005 [3]. Meningioma (MMA) is the most common primary brain tumor, of which 20% may display aggressive behavior [4] and develop early recurrences that require repeated surgeries, radiotherapy, or systemic treatments [5].

Extracellular vesicles (EVs) are membrane-enclosed nano-/micro-sized particles released by all cell types [6]. EVs can act as cell-to-cell communicators by delivering, inducing, and generating activity in both proximal and distal recipient cells [7,8]. Translation of EV-delivered mRNA by recipient cells as well as by the proliferation of glioma cells after treatment with glioma-derived EVs illustrates the consequential role of EVs as intercellular communicators in brain tumors [8,9]. Tumor cells release more EVs, which play a critical role in cellular communication in the tumor microenvironment, promoting tumor progression and invasion [10,11,12]. Glioma EVs have been shown to have major effects on glial cells such as astrocytes [13,14] and microglia [15]. Our published data demonstrated that GBM EVs possess unique characteristics and drive normal astrocytes toward a tumor-enhancing phenotype [13]. Many human diseases, including cancer, cardiovascular disease, and neurodegenerative diseases, have been found to have EV “signatures” [16,17,18].

One of the major challenges emerging in the field of EV utilization for clinical use is the lack of robust and reproducible methods for the isolation of a pure vesicular population. There is a lack of clear consensus for an optimal method of isolation of a pure EV population that is devoid of contamination with similar-sized vesicles of different origins. This is a major hurdle for the utilization of EVs and their components for clinical use, early detection, and screening of cancer.

Currently, EVs are isolated by differential centrifugation, membrane-based filtration, column chromatography, field-flow fractionation, and several affinity-binding techniques [19]. Due to the non-selective extraction of the current commercial EV kits, there is still no procedure that can isolate specific EVs from mixtures of different EVs in complex biofluid samples.

A key feature that would increase EV biomarker applicability would be the specific surface recognition of EVs released from a given cell or tissue source. Numerous protein markers and non-protein constituents such as lipids and glycoconjugates are likely highly specific as EV markers [20,21,22]. In this study, we have applied phage-displayed random peptide libraries to identify epitopes that are specific for GBM and MMA EVs. Phage-displayed peptides are capable of binding lipo-polysaccharides and lipoproteins [23,24], gangliosides [25,26], lipids [27], proteins [28], and other medically related targets of interest [29]. This flexibility in target discovery makes phage display a valuable technology to identify novel moieties that may reside on the surfaces of EVs in patients with brain tumors.

We have extensive experience in identifying epitopes/mimotopes of IgG antibodies in patients with multiple sclerosis [30,31,32]. Herein, we report the identification of phage peptides to GBM EVs by screening phage-displayed random peptide libraries (7 mer and 12 mer). We identified peptides that are specific to brain tumor EVs derived from both plasma and tumor cell lines. We further demonstrate that these peptides inhibited GBM EV-induced neuronal cytotoxicity. Identification of GBM EV-specific peptides provides a potential tool for biomarker development and strategies for effective therapeutics.

## 2. Results

EVs were derived from the individual plasma of GBM, MMA, and healthy controls. Plasma was also combined to generate three GBM pools and one MMA pool, due to limitations in the plasma samples. A pooled healthy donor plasma sample was obtained from Innovative Research, and additional individual healthy control plasma samples were purchased from Advanced Cure Project. The EVs were then purified from the pooled GBM, MMA, or HC plasma samples. Patient demographics are summarized in Supplementary Table S1. In this work, we utilized EV isolation methods that take advantage of particle density (ultracentrifugation), membrane topology (ExoEasy kits), and polymer-based precipitation (ExoQuick Exosome isolation kits).

### 2.1. Characterization of Brain Tumor Plasma-Derived Extracellular Vesicles

**Figure 1:**
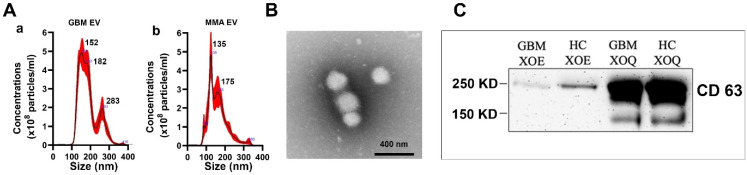
Characterization of EVs derived from plasma of patients with brain tumors. Plasma EVs were purified using ExoEasy and ExoQuick kits. (**A**) Nanoparticle Tracking Analysis showing examples of typical size and concentrations of the plasma EVs of GBM (**a**) and MMA (**b**) by ExoEasy. (**B**) Transmission electron microscope image showing ExoEasy purified GBM EVs, which are 100–200 nm diameters in size. (**C**) Western blots run on Native conditions demonstrated the presence of EV marker CD63 in GBM and HC plasma EVs. EVs were purified from plasma with ExoEasy (XOE) and ExoQuick (XOQ) followed by electrophoresis in Native gel. The blot was detected with mouse anti-human CD63 (1:1000). The CD63 proteins showed a much large size at 250 KD due to heavy glycosylation under native conditions, rather than the 25 KD under denaturing conditions. We found that CD63 protein was more abundant in Exo-Quick-purified EVs than in Exo-Easy-purified EVs.

We characterized EVs from pooled plasma, individual plasma, and tumor cell line media. EVs were analyzed for total protein content, size, and EV-associated markers. Total protein content was measured by micro-BCA. The EV size and particle concentrations (the number of particles per mL) were determined by Nanoparticle Tracking Analysis (NTA) using a Nanosight device. Representative NTA plots for GBM- and MMA-pooled plasma-derived EVs are shown in Figure 1A, with an average diameter of about 150 nm and 10^8^ particles/mL of plasma. We further characterized GBM plasma EVs with transmission electron microscopy (TEM) after negative staining. A representative image of GBM EV is shown in Figure 1B. Additionally, Western blots were used to confirm that EV-associated surface markers were presented in the brain tumor plasma EVs. The EVs were lysed by 8 M urea followed by Native-PAGE and Western blotting to detect the classical EV marker CD63. We showed that both GBM and HC plasma-derived EVs expressed CD63 protein about 250 kDa in size, because the heavily glycosylated CD63 protein runs much slower in native conditions than the denatured CD63 protein, which is about 25 KD. The appearance of a much large sized CD63 protein under the native gel condition has been reported before [33,34,35]. We noted the CD63 protein levels were lower in EVs isolated by ExoEasy (XOE) methods as compared to that isolated by the ExoQuick kit (XOQ), although the same concentration of EVs was loaded (Figure 1C).

### 2.2. Screening Phage-Displayed Random Peptide Libraries with Brain Tumor EVs Identified High-Affinity Phage Peptides with Identical and Homologous Sequences

We applied a combination of Phage-Displayed Random Peptide Libraries (7-mer and 12-mer) for peptide selection. We carried out a total of five rounds of independent phage library panning experiments with the traditional panning method (New England Biolab) and an ultra-fast peptide selection procedure [36]. We extended the panning process from two to five rounds of biopanning to ensure the selection of high-affinity phage peptides. After five rounds of panning, we selected the phage, which showed higher bindings to panning EVs compared to Healthy Control EVs and BSA for further characterization, including DNA sequence, homology alignments, and large-scale amplification for dose–response ELISA and immunoprecipitations.

We identified a total of 44 peptides by brain tumor EVs, of which 34 peptides are unique (Supplementary Table S5). Although we used an equal mixture of 7-mer and 12-mer peptide libraries for the screening, most of the positive peptides were 12 mers (90%) rather than 7 mers (10%), indicating that longer peptides were more likely to bind to the EV targets. We suspect that the longer peptides display more surface area capable of interacting with EV epitopes compared to 7-mer peptides.

Nine peptides were identified multiple times (two to four times across targets). From GBM pooled plasma EVs (ppEV), we identified one over-represented sequence AEAWTGFSASGV (4/8, 50%). We carried out multi-sequence alignments (https://www.ebi.ac.uk/Tools/msa/clustalo/, accessed on 21 October 2021). We demonstrated that identical and homologous peptide sequences were selected by individual brain tumor extracellular vesicle groups (Supplementary Table S6).

To see if these peptides share sequence homologies with proteins of interest, we searched the protein database using BLSAT (https://blast.ncbi.nlm.nih.gov/Blast.cgi, accessed on 21 October 2021) with representative peptides from each EV group. Because of the short length of the peptides, we focused on human protein targets only. In Supplementary Table S7, we listed the BLAST-identified protein homologs to representative peptide sequences. Many of these homologous sequences are related to immunoglobulin heavy and light chains, glycoproteins, and Ig-domain-containing proteins. For example, the peptide sequence VHWDFRQWWQPS (four out of nine sequences), which was selected by MMA cell line EVs, showed homology sequences of anti-HIV-1 immunoglobulin heavy chain variable region (5/7 identical peptides), and immunoglobulin heavy chain junction region (5/7 identical peptides).

### 2.3. Phage ELISA Assays Demonstrated the Specific Bindings of Phage Peptides to Corresponding Panning Brain Tumor EVs

**Figure 2:**
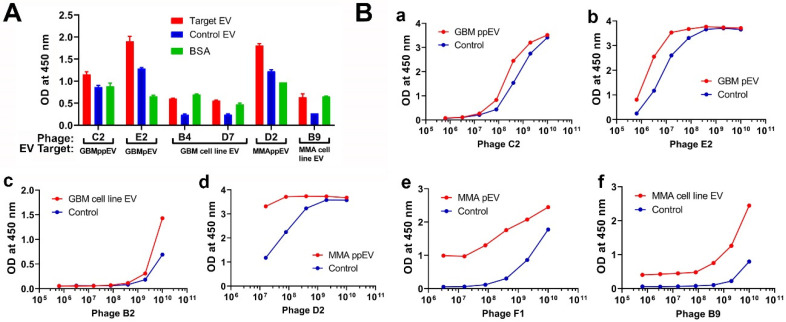
ELISA demonstrates that phage peptides selected by brain tumor extracellular vesicles are specific to panning EVs. (**A**) Direct ELISA shows that phage peptides are specific to panning brain tumor EVs. The target EVs include GBM and MMA ppEVs (from pooled plasma), pEVs (from individual plasma), and tumor cell line EVs. EVs coated on a high-binding ELISA plate (50 mg/mL) were incubated with corresponding phage (2 × 10^9^ pfu/well). Bound phage peptides were detected by mouse anti-M13 pIII-HRP antibody (1:5000 dilution) followed by TMB substrate color detection. Each representative phage peptide (labeled in letters) was shown for OD values to corresponding panning EVs (target EV), control EVs, and BCA. (**B**) Dose–response ELISA demonstrating specific bindings of phage peptides to panning brain tumor EVs. GBM (**B**-**a**–**c**) and MMA EVs (**B**-**d**–**f**) (pooled plasma, individual plasma, and tumor cell line EVs) at 50 mg/mL were directly coated onto wells of ELISA plates, followed by incubation with corresponding phage peptides in serial 5-fold dilutions starting with 1 × 10^10^ pfu/well. Detection was the same as described above. HC plasma EVs were used as controls for brain tumor plasma EVs (**a**,**b**,**d**,**e**), and normal human astrocyte EVs were used as controls for brain tumor EVs (**c**,**f**).

We amplified and purified representative phage and carried out an ELISA to determine if these phage peptides have specific reactivities to panning tumor EVs compared to control EVs and BSA. EVs were coated onto ELISA wells overnight, followed by incubation with corresponding phage peptides. Phage peptides showed higher bindings to panning brain tumor EVs from both GBM and MMA plasma/cell lines compared to control EVs and BSA for each phage peptide tested (Figure 2A). We used BSA as a negative control as serum albumin is frequently associated with plasma EVs.

To further demonstrate the specificity of phage binding to target EVs, we carried out a dose–response phage ELISA with representative phage peptides selected from GBM pooled plasma EVs, MMA pooled plasma EVs, individual GBM or MMA EVs, or tumor cell line EVs. For each phage peptide, we made a serial 5-fold dilution and added it to target and control EV coated wells. Bound phages were detected with HRP conjugated anti-pIII M13 antibodies. The results are shown in Figure 2B. Higher bindings of phage to corresponding EVs but not control EVs are shown with increased phage titer for all phage peptides tested, suggesting that these peptides are specific to the panning brain tumor EVs. Higher OD readings at low phage titers are presumably indicative of more avid phages, but this may be an effect of the target moiety on EVs.

### 2.4. Transmission Electron Microscopy Analysis of PHAGE–EV Co-Precipitation

**Figure 3:**
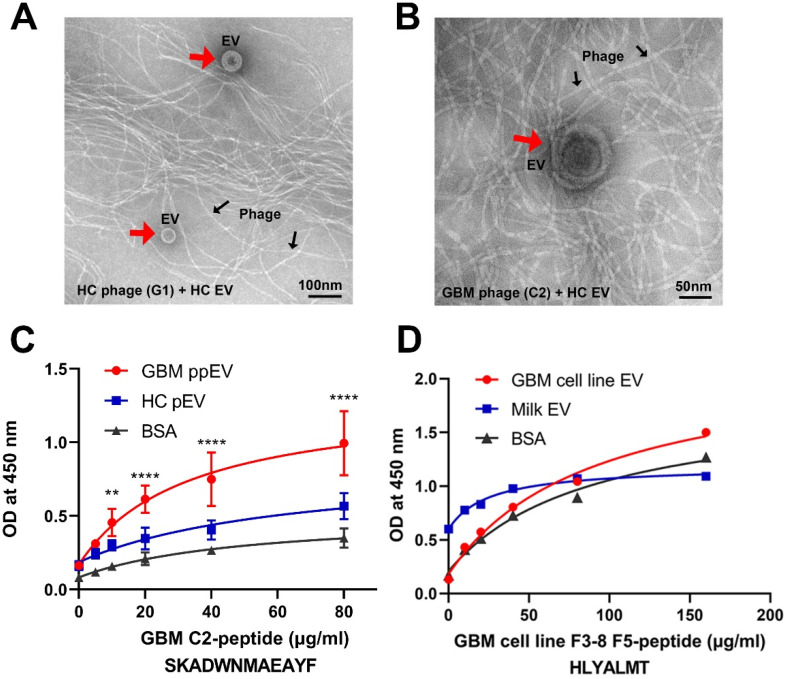
High-affinity bindings of phage peptides to GBM EVs are demonstrated by electron microscopy and synthetic peptide ELISA. (**A**,**B**) Electron microscopic images showing EVs precipitated by phage peptides. GBM EV phage C2 and HC EV phage G1 (2 × 10^10^ pfu) were incubated with HC-EVs (100 µg) overnight at 4 °C. The EV–phage complexes were precipitated by PEG 8000/NaCl, dissolved in PBS followed by transmission electron microscopy. (**A**) HC G1 phage and HC EVs. (**B**) GBM C2 phage and HC EVs. The EVs (red arrows) precipitated by M13 phage (black arrows) show typical spherical shapes with size ranges of 50–200 nm in diameter. Notice that samples in image (**A**) are 1:20 dilution and samples in (**B**) are original with no dilution, indicating that there are fewer HC EVs precipitated by GBM phage C2 compared to HC EVs by HC phage. (**C**,**D**) ELISA shows dose responses of synthetic peptide binding to corresponding EVs. EVs (20 µg/mL) and BSA protein (20 µg/mL) were coated onto wells of ELISA plates overnight at 4 °C. Synthetic biotinylated peptides in 2-fold serial dilutions were added to corresponding wells. Bound peptides were detected by NeutrAvidin HRP (1:10,000) followed by the TMB color reaction. (**C**) GBM plasma EV C2 peptide had significantly higher bindings to corresponding GBM ppEV compared to HC-EV or BSA (** *p*< 0.01, **** *p* < 0.0001). (**D**) GBM tumor cell line EV peptide demonstrated higher bindings to corresponding GBM cell line EVs compared to bovine milk EVs or BSA.

To test whether phage surface-displayed peptides can recognize and bind to the corresponding target EVs, we performed phage–EV co-precipitation followed by transmission electron microscopy (TEM) analysis. As proof of principle study, we incubated GBM EV phage C2 or HC EV phage G1 with HC-EVs overnight at 4 °C, followed by PEG 8000 precipitation and TEM analysis. The TEM images showed that EV particles (red arrowheads) were present together with a large number of filamentous phages (black arrows). Figure 3A shows the results for HC phage incubated with HC EV after samples were diluted at 1:20 due to the high density of particles. We applied the same amount of HC EVs with GBM phage C2 to test the cross-reactivity. Figure 3B shows that GBM phage C2 can precipitate HC EV, but with less binding efficiency compared to HC phage G1 precipitated HC EVs in Figure 3A (undiluted vs. 1:20 diluted samples of the precipitated EV).

### 2.5. Affinity Binding Analysis of Synthetic Peptides Confirmed Specific Bindings to Brain Tumor EVs

To further characterize the binding specificity of the EV-selected phage peptides, we picked high binding phages based on the ELISA results and synthesized representative peptides with N-terminal biotinylated modifications. The purities of peptides were all above 95%. We carried out a dose–response ELISA for the peptides. The peptides were dissolved in DMSO or DMF due to hydrophobic features. EV-coated ELISA wells were incubated with corresponding biotinylated peptides in serial two-fold dilutions. Bound peptides were detected with NeutrAvidin-HRP. As examples of the peptide ELISA, we showed that the GBM plasma EV peptide C2 bound to the EVs in a dose-dependent manner (Figure 3C). Similarly, the GBM tumor cell line EV peptide F5 had dose-dependent bindings to panning EVs compared to controls (Figure 3D).

**Figure 4:**
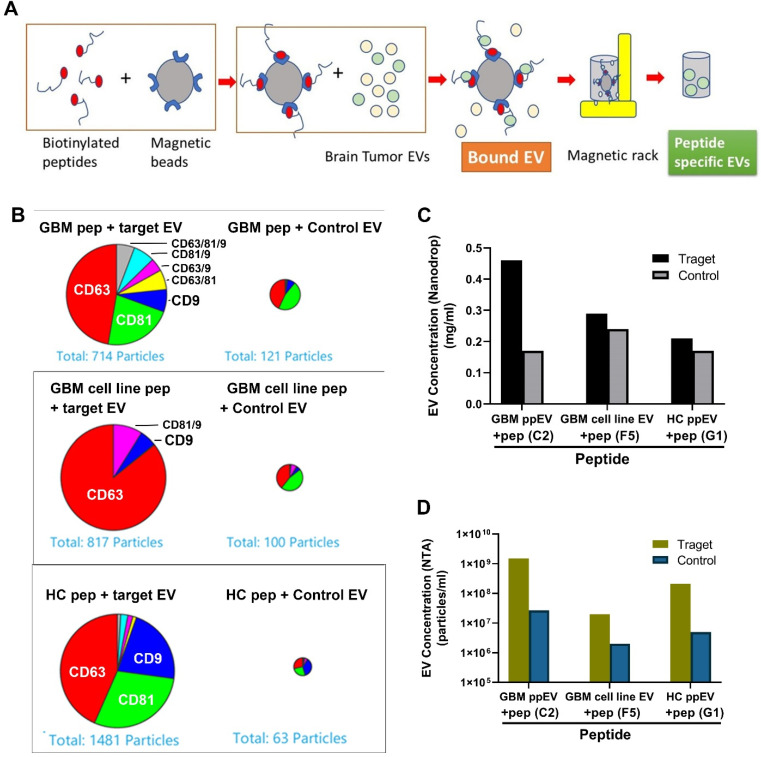
A higher number of GBM EVs was precipitated by EV-specific synthetic peptides. (**A**) Schematic drawing of EV precipitation with peptide magnetic beads. The biotinylated peptides bound to streptavidin linked Dynabeads were incubated with EVs at 4 °C overnight, and EVs were eluted with low pH buffer and neutralized with 1 M Tris-HCl (pH 10). (**B**) ExoView analysis demonstrated that higher amounts of EVs were affinity-precipitated by corresponding peptides compared to control peptides. GBM plasma EVs (top panel), GBM tumor cell line EVs (middle panel), and HC plasma EVs (lower panel) were precipitated by corresponding peptides and control peptides, followed by analysis with ExoView. About 5- to 20-fold more target EVs were precipitated by peptides compared to control EVs. (**C**) Higher target EV concentrations precipitated by specific peptides compared to control EVs. (**D**) NTA showed that higher target EV concentrations were precipitated by specific peptides compared to control EVs. Specific peptides can capture 10–50 times more target EVs than control EVs.

We further carried out EV affinity purifications with representative biotinylated peptides. We saturated streptavidin-linked magnetic beads (Dynabeads) with the biotin EV peptides, followed by incubations with target EVs. The bound EVs were eluted with a low pH buffer followed by neutralization (Figure 4A). To obtain a more detailed characterization of the peptide-precipitated EVs, we used the ExoView^®^ Tetraspanin Assay (NanoView Biosciences) for quantification and phenotyping. EVs were analyzed using single-particle interferometric reflectance image sensing by the ExoView R100 platform. The CD63, CD81, and CD9-positive immuno-captured EVs from the eluted EV sample were imaged and counted.

Results showed a higher number of tetraspanin-positive vesicles present in the target EV samples (panning EVs) compared to control non-panning EVs (Figure 4B). Comparing target vs. non-target EV pull-down, about six times more GBM plasma EVs were precipitated by the GBM peptide compared to control EVs. Similarly, eight times more target EVs by GBM cell line peptide and 23 times more HC EVs by HC peptide were precipitated than non-target EVs. The most abundant EV surface protein is CD63 in all three EV subpopulations analyzed. Similarly, the results of total protein concentrations of the eluted EVs by Nanodrop showed that higher amounts of target EV (e.g., GBM-C2 peptide and GBM-ppEV) were precipitated compared to control EVs (Figure 4C). In addition, NTA data demonstrated that the peptides precipitated 10–50 times more target EVs than controls (Figure 4D).

### 2.6. GBM EV Caused Necrotic Cell Death in Neurons

**Figure 5:**
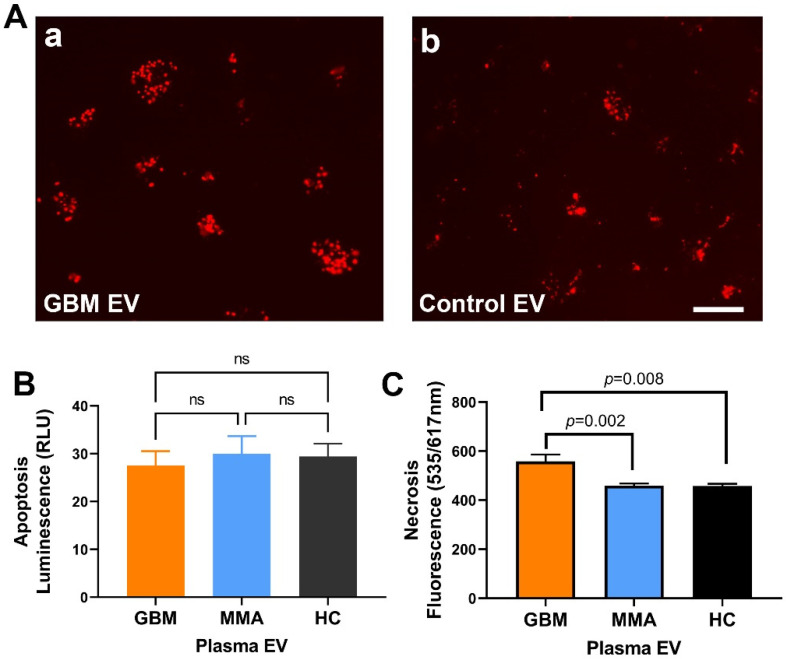
GBM EV induced necrotic cell death in neurons (**A**). Fluorescent microscopy shows higher neuronal death induced by GBM EVs. SH-SY5Y cells were treated with EVs for one hour. Cell death was monitored by propidium iodide. A higher number of neuron death was seen in GBM EVs (**A**-**a**) as compared to control EVs from an epithelial cell line (**A**-**b**). (**B**). GBM EV did not cause apoptosis in SH-SY5Y cells. SH-SY5Y cells were treated with GBM, MMA, or HC EVs for four hours, and apoptosis was measured by luminescence. Only minimal luminescence signals were detected in GBM, MMA or HC EV-treated cells, and there are no significant differences among different EVs (n.s., *p* > 0.1, n = 6 for GBM EV, n = 8 for MMA EV, n = 5 for HC EV). (**C**). GBM plasma EVs induced higher necrosis as compared to control EVs. SH-SY5Y cells were incubated with plasma EVs (2 mg/mL, GBM EV=14; MMA EV = 8; HC EV = 8) for 4 h. The necrotic cell death was monitored by PI red fluorescent reading. There were significantly higher levels of necrosis in GBM-EV-treated neurons compared to MMA EV and HC-EV-treated cells (*p* = 0.002 and *p* = 0.008, respectively). Scale bar = 200 mm for (**A**).

We previously reported that GBM-derived EVs drive normal astrocytes toward a tumor-enhancing phenotype [13]. To see if GBM EVs produce neuronal cytotoxicity, we used the neuroblastoma SH-SY5Y cell line as neuronal cell surrogates. We incubated GBM cell line-derived EVs and control cell line EVs (from an epithelial cell line) in SH-SY5Y cells plus normal human serum for two hours and examined cell death using propidium iodide (Figure 5A). In cells treated with GBM EVs (Figure 5A-a), a higher number of dead cells were present compared to control EV-treated cells (Figure 5A-b). To test if GBM EV can induce apoptotic cell death, we measured the apoptosis by luminescence assay. There were only minimal levels of luminescence in SH-SY5Y cells after treatment with GBM, MMA, HC EVs for 4 h. There were no significant differences in apoptosis in any of the EV-treated cells (Figure 5B, n = 6 for GBM EV, n = 8 for MMA EV, n = 5 for HC EV). This observation indicated that apoptosis was not involved in EV-induced toxicity. To further uncover the mechanism of GBM EV induced cytotoxicity in neurons, we measured necrosis using PI red fluorescence (535/617 nm) in SH-SY5Y cells treated with EVs purified from individual plasma of 14 GBM patients, 14 MMA patients, and 8 healthy controls. We demonstrated that GBM plasma EVs produced significantly higher levels of necrosis compared to MMA EVs (*p* = 0.002) and healthy control EVs (*p* = 0.008) (Figure 5C). The results suggest that GBM EV can cause necrotic cell death in neurons.

### 2.7. GBM EV-Specific Peptides Inhibit Neuronal Cytotoxicity Caused by the GBM EVs

**Figure 6:**
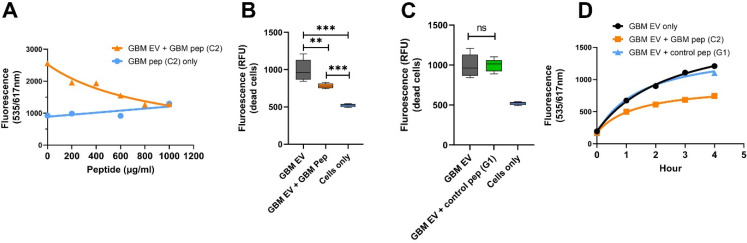
GBM EV-specific peptides inhibit neuronal cytotoxicity induced by the GBM EVs. (**A**). GBM EV-specific peptide inhibits EV-induced neuronal cytotoxicity in a dose-dependent manner. SH-SY5Y cells were incubated with GBM pooled plasma EVs at 20 mg/mL plus 5% Normal Human Serum. GBM EV-specific peptide C2 in various amounts was added to cells at the same time as the EV treatment. Cell death was monitored by ethidium homodimer-1 (red fluorescent reading). With 1000 mg/mL peptide, the GBM EV peptide nearly completely blocked the GBM-EV cytotoxicity after 4-h treatment. Cells with peptides (C2) alone did not produce cytotoxicity. (**B**,**C**). GBM EV-specific peptides (**B**), but not the control peptides (**C**) show time-dependent inhibition of EV induced neuronal death. Neuroblastoma SH-SY5Y cells were treated with GBM EVs plus peptides, and cytotoxicity was monitored for 4 h. GBM EV-specific peptide C2 inhibited GBM-EV induced cytotoxicity (GBM EV + GBM Pep), but not the control peptide to HC EV (G1) (GBM EV + control pep G1) (** *p* < 0.01, *** *p* < 0.001). (**D**) Time course of GBM-C2 peptide for the inhibition of GBM-EV cytotoxicity. SH-SY5Y cells were treated with GBM EVs at 20 mg/mL plus 5% Normal Human Serum. The GBM-C2 peptide (1000 mg/mL) or the HC-G1 peptide (as control, 1000 mg/mL) was added to cells during the same time as EV treatment. The cell death was monitored by propidium iodide red fluorescent reading once per hour. Results showed that the GBM peptide can inhibit GBM EV-induced cell death.

To investigate if the GBM EV-specific peptides can inhibit GBM EV-induced neuronal toxicity, we incubated GBM EV peptide C2 (2-fold serial dilutions) and GBM EVs in SH-SY5Y cells for 4 h with fluorescent intensity measurements. GBM EV peptide C2 prevented neuron death in a dose-dependent manner, but the cells incubated with peptide alone did not show cytotoxicity (Figure 6A). At the dose of 1 mg/mL of GBM-C2 peptides, the neuronal cytotoxicity was mostly abolished, suggesting that the peptide may block the access of complement components required for cell lysis. To confirm that the peptide inhibition is specific, we treated SH-SY5Y with GBM plasma EVs (n = 6), together with GBM EV peptide (Figure 6B) or control peptide HC G1 (Figure 6C) for 4 h. Cells treated with GBM EV peptide showed significantly lower levels of cytotoxicity compared to GBM EV treated cells (Figure 6B, ** *p* < 0.01). However, no reduction in neuronal toxicity was observed in cells treated with control peptides (Figure 6C). We also analyzed the time course of peptide inhibition for GBM EV-induced cell death (Figure 6D). We found the GBM-C2 peptide can greatly reduce the GBM-EV induced cytotoxicity, while the HC-G1 control peptide did not reduce GBM-EV cytotoxicity (Figure 6D).

## 3. Discussion

A specific EV biomarker to identify brain tumors is needed for early diagnosis and monitoring treatment response. Currently, EV isolation is largely accomplished with antibody-binding to EV surface markers [37]. In this study, we utilized an unbiased approach to identify peptide ligands for high-affinity binding to EVs from brain tumor patients using phage-displayed random peptide libraries. We identified multiple high-affinity phage peptides that are specific to GBM, MMA, or tumor cell line EVs. Significantly, these peptides can act as inhibitors preventing brain tumor EV-induced neuronal cytotoxicity.

We screened a combination of two phage-displayed peptide libraries with these tumor EVs using both traditional panning methods and an improved fast panning protocol [36] and identified a total of 44 peptides from GBM, MMA, or cell line EVs. Identical and homologous peptides were identified in each EV panning group, suggesting that these peptides are specific to the corresponding EVs. Furthermore, the results of phage ELISA, synthetic peptide ELISA, and peptide affinity EV precipitations confirmed that these peptides are specific to panning brain tumor EVs, indicating that they may represent unique epitopes/mimotopes of brain tumor EVs. Although, to a lesser extent, phage ELISA showed that tumor EV peptides had reactivities to control EVs, suggesting that brain tumor EVs shared epitopes with healthy plasma EVs. Further investigations are needed to elucidate the targets of the peptides.

Although both 7-mer and 12-mer peptide libraries (1:1) were used for the panning experiments, most of the peptides selected were 12 mers (Supplementary Table S5). We speculate that longer peptides (12 mer) may form secondary structures that bind GBM EVs with higher affinity, suggesting that these peptides may represent conformational epitopes for the extracellular vesicles. Future studies will include libraries with longer peptides (15 mer, for example)

Through phage peptide/EV and synthetic peptides/EV pull-down experiments, we confirmed that these peptides can directly bind and capture their target EVs in solution. These peptides could develop novel tools for efficiently purifying brain tumor EVs and one can expand phage peptide identification by applying EVs from diverse sources of plasma, including patients with other neurological disorders and healthy donors. These unique peptides may provide novel tools for understanding the roles of EVs in the disease and for strategies of standardizing methods for the isolation of EVs.

Current EV isolation methods indiscriminately extract all circulating EVs in a biofluid, regardless of their cellular origin. Affinity-based EV isolation methods have been developed [38,39] using antibodies to EV surfaces markers CD9, CD63, CD81, and Hsp70 [40,41]. However, these methods cannot separate the tumor cell-produced EVs from healthy cell-produced EVs. Our results of peptide-bound EVs may be unique for the given tumor types and therefore may provide clues to the biochemical and genetic components of tumor-derived specific EVs as biomarkers or biological agents. Our identification of brain tumor EV-specific peptides provides opportunities to isolate unique tumor EV subpopulations.

Synthetic combinatorial peptide libraries have been used successfully to identify bioactive peptides, including antimicrobial peptides [42], opioid receptor antagonists [43], ligands for cell surface receptors [44], and T cell epitopes [45]. Here, we demonstrated that the brain tumor EV-specific peptides can be used to inhibit EV-induced neuronal cytotoxicity in a dose-dependent manner, suggesting that these peptides can be exploited as novel inhibitors for preventing tumor pathogenesis. The identification of brain tumor EV peptides expands the repertoire of bioactive peptides [46], which provides novel tools for EV characterization and for understanding EV-mediated disease pathogenesis.

## 4. Materials and Methods

### 4.1. Human Samples

The study has been approved by the Colorado Medical Institutional Review Board (COMIRB, #13–3007). The blood and tumor samples were collected from brain tumor patients during the surgery. The plasma samples were processed and stored at the Nervous System Biorepository at the Department of Neurosurgery, University of Colorado Anschutz Medical Campus. In our clinical practice, for patients with newly diagnosed high-grade gliomas, at least one-third of those patients are on some form of steroids (such as dex) pre-operatively. Over half of those patients are on AEDs such as Keppra. For patients with recurrent high-grade gliomas, approximately 75% of the patients are on steroids and nearly 100% on AEDs before their next surgeries. These drugs have known anti-inflammatory properties, suggesting that at the time of blood collection (at the start of surgery), the EVs obtained in blood are not likely to reflect an inflammatory state. A pooled healthy donor plasma sample was obtained from Innovative Research (Novi, MI, USA, ISERAB-100 mL), and its EVs were purified and used for initial phage screening. Material tests negative for HBsAG, HIV-1/2, HIV-1Ag, anti-HCV, HCV-Nat, HIV-1 NAT, ALT, West Nile and Zika virus NAT, and Syphilis per FDA Regulations. Additional individual healthy control (HC) plasma from 20 subjects were obtained from the Accelerated Cure Project (Waltham, MA, USA, https://www.acceleratedcure.org/, accessed on 21 October 2021).

### 4.2. Cell Lines

Brain tumor cell lines were derived from surgically resected primary intracranial tumors from patients receiving treatment at the University of Colorado Hospital [13]. Normal human epithelial cells were obtained from healthy donor urine and a cell line was established as described [13]. For EV purification, the serum-free culture media was used, and the spent media was collected and stored at 4 °C.

### 4.3. Extracellular Vesicle Isolation Using the Ultracentrifugation Method

The spent media was cleared through centrifugation at 17,000 rpm for 20 min. The purification procedure was as described [47].

### 4.4. Extracellular Vesicle Isolation Using ExoEasy and ExoQuick Commercial Kits

For the ExoEasy Qiagen kit (Qiagen #76064, Redwood City, CA, USA), plasma was pooled, and each pool contained an equal volume of plasma from ten patients, due to limited plasma samples. There were 3 GBM pools, 1 MMA pool, and 1 HC pool. Purified EVs were stored at 4 °C for the duration of the project. The fat-free bovine milk was dissolved in PBS and used for EV purification with an ExoEasy kit, termed as milk EVs. For plasma EV purification with the ExoQuick kit (SBI, EXOQ20A, Palo Alto, CA, USA), we followed the manufacturer’s instructions. Briefly, 200 µL of plasma was cleared by centrifugation at 3000× *g* for 15 min at 4 °C. The supernatant was mixed with 50 µL of ExoQuick solution and incubated at 4 °C for 30 min with rotation, followed by centrifugation at 1500× *g* for 30 min. The pellets were dissolved in 200 µL of PBS, aliquoted, and stored at −80 °C.

### 4.5. EV Characterization

Total protein concentration from the isolated EVs was determined using the Pierce BCA kit (Thermo #23227, Waltham, MA, USA) according to the manufacturer’s instructions. The EV size and quantity were determined by Nanoparticle Tracking Analysis (NTA) using Nanosight device NS300 (Malvern, UK), software Version: NTA 3.2 Dev Build 3.2.16; Malvern P analytical for the analysis. The experiments were repeated at least 3 times to obtain representative results.

### 4.6. Western Blots

EVs were lysed in 16 M urea for 1 h at 37 °C. The protein lysates (10 µg/lane) were mixed with Laemmli buffer without β-mercaptoethanol. The proteins were separated on 10% Non-denature Criterion Tris-HCl protein gel (Biorad 3450009, Hercules, CA, USA) under the native condition with Tris/Glycine running buffer without SDS and transferred to a nitrocellulose membrane. The blot was blocked with 1× Casein and probed for EV marker CD63 (Abcam, Cambridge, United Kingdom, ab59479, mouse anti-human CD63, diluted 1:1000 in 1× blocking buffer) overnight at 4 °C. The HRP-conjugated goat anti-mouse secondary antibody (Thermo #31430, 1:20,000, Waltham, MA, USA) was incubated for 1 h at room temperature. The blots were developed with SuperSignal™ West Femto Maximum Sensitivity Substrate (ThemoFisher #34095, Waltham, MA, USA), and the signal was detected with a Cell Biosciences FluorChemQ MultiImage III (San Jose, CA, USA).

### 4.7. Phage-Displayed Random Peptide Library Screening

Ph.D.™-7 and Ph.D.™-12 libraries (New England Biolabs, #E8110S, Ipswich, MA, USA) were panned with a slightly modified protocol. We combined two phage peptide libraries for the identification of brain tumor EV-specific peptides. The panning procedure, as well as the characterization of positive phage peptides, were described previously [31,32]. Briefly, ELISA wells (Thermo #436110, Waltham, MA, USA) were coated with panning EVs (individual or pooled GBM EVs, individual or pooled MMA EVs, tumor cell line EVs, each at 50 mg/mL, 100 µL) in bicarbonate buffer (0.1 M NaHCO3, pH 8.6) overnight at 4 °C. Unbound EVs were washed off with Tris-buffed saline (TBS) + 0.1% Tween 20 (TBST). Wells were blocked with 3% BSA. The combined peptide libraries in equal proportions (1.5 × 10^11^ pfu in 100 µL TBST) were added to coated wells and incubated for 1–3 h at room temperature with gentle rocking or overnight at 4 °C with gentle rocking. EV bound phage was eluted with 100 µL 0.2 M glycine, pH 2.2/0.1% BSA for 10 min followed by neutralization with 15 µL of 1 M Tris, pH 9.5. The eluted phage was titered and amplified. A streamlined protocol was used to determine phage peptide specificity after affinity selection [30]. Positive phage clones to panning EVs were confirmed by phage ELISA with BSA as control. DNA from positive phage clones was purified and sequenced.

#### 4.7.1. Phage Titering, Amplification, and DNA Sequencing

We followed the previous protocol [30] for determining phage pfu, large-scale phage amplification, and DNA sequencing.

#### 4.7.2. DNA Sequencing of Individual Phages

Single-stranded phage DNA was purified using the QIAprep Spin M13 Kit with a QIAvac 24 vacuum manifold (Qiagen). The phage DNA was suspended in 50 µL of 10 mM Tris–HCl, pH 8.5, and the DNA (8 µL) was sequenced with M13 sequencing primer—96 gIII (New England BioLab) at the University of Colorado Cancer Center DNA sequencing core. Multi-sequence alignments were performed with the online Cluster Omega tool: https://www.ebi.ac.uk/Tools/msa/clustalo/, accessed on 21 October 2021. BLAST searches were performed in human protein databases.

### 4.8. Phage ELISA with Anti-M13 Antibody

The 96-well ELISA plates (Thermo #436110, Waltham, MA, USA) were coated with EV targets at 50 mg/mL overnight at 4 °C. The unbound target was washed off with TBST, followed by incubation with a blocking buffer (TBS with 1% BSA) for 1 h at room temperature. After washing, respective phages were added and incubated overnight at 4 °C. Bound phage was detected with HRP-conjugated mouse anti-phage M13 pIII antibody (NEB #E8033S, Ipswich, MA, USA, 1:5000 in 1× TBS/3% BSA) followed by TMB color reaction. The plate was read in a microplate reader (Biotek Synergy, Santa Clara, CA, USA) at 450 nm.

### 4.9. Peptide Synthesis

The peptides were synthesized with biotinylated modification at the C terminal (10 mg, >95% purity) by Vivitide (Gardner, MA, USA, www.vivitide.com, accessed on 21 October 2021). The synthesized peptides were dissolved in DMSO or DMF and aliquoted and stored at −80 °C before use.

### 4.10. Synthetic Peptide ELISA

The 96-well ELISA plates with 100 µL (20 mg/mL) of target EVs overnight at 4 °C. The wells were blocked (TBS with 1% BSA) followed by the addition of biotinylated peptides (0–160 mg/mL diluted in 1× TBS) and incubated overnight at 4 °C. Bound peptides were detected with NeutrAvidin-HRP (Thermo #31001, Waltham, MA, USA, 1:10,000 in TBS) for 2 h at room temperature with shaking. TMB color detection was performed as described above.

### 4.11. Negative Staining of EVs and Visualization via Transmission Electron Microscopy (TEM)

The negative staining and TEM observation of EV was carried out at the Electron Microscopy Center of the University of Colorado, Anschutz School of Medicine. For each sample, 2 µL was applied to a copper mesh grid coated with formvar and carbon (Electron Microscopy Sciences, PA, USA) for two minutes and then gently blotted off with a piece of Whatman filter paper. The grids were rinsed with transfers between two drops of MilliQ water, blotting between each transfer. Finally, the grids were stained with two drops of a 0.75% uranyl formate solution—a quick rinse with the first drop followed by 20 s of staining in the second drop. After blotting, the grids were allowed to dry for at least 10 min. Samples were imaged on an FEI Tecnai G2 Biotwin TEM (ThermoFisher, Waltham, MA, USA) at 80 kV with an AMT side-mount digital camera (AMT Imaging, Woburn, MA, USA).

### 4.12. Phage–EV Co-Precipitation

Representative phage (1 × 10^12^ pfu/mL) were incubated with corresponding and control EVs (100 mg) for 2 days at 4 °C with rocking. The phage–EV complexes were precipitated with 1/4 volume of 20% PEG-8000/2.5 M NaCl at 4 °C overnight followed by centrifugation at 14,000× *g* for 50 min at 4 °C. The pellets were resuspended in 50 mL of TBS for transmission electron microscopy.

### 4.13. Peptide Affinity Precipitation of GBM EVs

We incubated 100 mL of Dynabeads M-280 Streptavidin (10 mg/mL, Invitrogen, #112050, Waltham, MA, USA) with biotinylated EV peptides (10 mg/mL) for 2 h at room temperature. The peptide-coated beads were separated with a magnet, followed by 3 washes with 0.1% BSA in PBS. Peptide-bound beads were resuspended in 100 mL of PBS and mixed with 500 mg (in 500 mL PBS) of EVs followed by incubation overnight at 4 °C. The EV-bound beads were collected eluted with 50 mL of Pierce IgG Elution Buffer (Pierce #21004, Waltham, MA, USA) and mixed immediately with 5 mL of neutralization buffer (1M Tris-HCl, pH 10).

The ExoView R200 platform (NanoView Bioscience, Brighton, MA) was used to characterize the precipitated GBM EV subpopulations. Briefly, 25 µL of EV solutions (10^6^–10^7^ particles/mL) was mixed with 25 µL of binding buffer. The mixture was added to the ExoAssay chip in a 24-well plate for overnight coating. After washes followed by incubation with blocking buffer and antibody solution, which included anti-CD9, anti-CD81, and anti-CD63 antibodies. The chip was washed and loaded to the ExoView R200 platform for reading. The data were analyzed by ExoView Software Suite.

### 4.14. Assessing EV Cytotoxicity in Neuroblastoma SH-SY5Y Cells

SH-SY5Y neuroblastoma cells (ATCC, #CRL-2266, Manassas, VA, USA) cells were maintained in DMEM with 10% FBS and 1× Penicillin-Streptomycin and plated on a 0.1% gelatin-coated 10 cm tissue culture plate. Cells at passage numbers 5–10 were used for cytotoxicity assays. For live-cell imaging, the cells were seeded in 24-well plates and maintained up to 7 days before treatment when the cell density was about 70% confluency. For EVs cytotoxicity assays, cells were plated at 20,000/cm^2^ in a 96-well tissue culture plate (BD Falcon #353219, San Jose, CA, USA). EV treatment mixtures included various amounts of EVs, synthetic peptides, 5% Normal Human Serum as a source of complement (Complement Technology Inc, Tyler, TX, USA), 1× RealTime-Glo Annexin V Apoptosis reagents (Promega JA1011, Madison, WI, USA), or 1× cell viability assay reagents (2 mM ethidium homodimer-1 or 5 mg/mL of propidium iodide, Waltham, MA, USA), and culture medium to a total of 100 µL per well. Cells were washed once with a fresh warm culture medium before treatment. The treatment mixture was added to SH-SY5Y cells, and the cytotoxicity was measured by relative luminescence (for apoptosis) or green/red fluorescent signals (for live cells and necrosis) every hour with a plate reader (535/717 nm, Biotek Synergy H4 hybrid reader, software version Gen5 3.09, Santa Clara, CA, USA) for 4 h.

### 4.15. Data Analysis

Experiments were repeated at least once. Data were analyzed using Prism software (San Diego, CA, USA). One way or two-way ANOVA analysis was performed followed by pairwise multiple comparisons with Tukey’s method to show the significant differences between treatments and groups. Data were presented as mean ± SEM (or SD). Graphs were plotted with GraphPad Prism 8.0.

## Data Availability

Data are available upon reasonable request.

## Supplementary Materials

The following supporting information can be downloaded at: https://www.mdpi.com/article/10.3390/ijms23137200/s1.
