# Supplementary material for: Glioblastoma Extracellular Vesicle-Specific Peptides Inhibit EV-Induced Neuronal Cytotoxicity
Source: Int J Mol Sci. 2022 Jun 28;23(13):7200. doi: 10.3390/ijms23137200 (PMC9266738; doi:10.3390/ijms23137200)
Supplement: Supplementary file 1 [file ijms-23-07200-s001.zip › ijms-1733364-supplementary.pdf]

Glioblastoma tumor extracellular vesicle specific peptides inhibit EV-induced neuronal cytotoxicity

Wenbo Zhou[#], Julia Craft[#], Alex Ojemann, Luke Bergen, Arin Graner, Aitana Gonzales, Qianbin He, Timothy Kopper, Marie Smith, Michael W. Graner*, Xiaoli Yu*[§]

Department of Neurosurgery, University of Colorado Anschutz Medical Campus, Aurora, CO, 80045.

Supplemental Tables

1. **Table S1: Subject demographics (summary)**
2. **Table S2: GBM patient details**
3. **Table S3: MMA patient details**
4. **Table S4: Healthy control details**
5. **Table S5: List of peptides identified**
6. **Table S6: Peptide sequence alignments**
7. **Table S7: List of peptides with homologies to known proteins**
8. **Table S8: List of synthetic peptides**

Supplemental Table S1. Summary of subject demographics

Diagnostic	Glioblastoma (GBM), WHO Grade IV	Meningioma (MMA), WHO Grade I	Healthy Controls (HC)*
Subject (n)	40	40	20
Male (n)	28	13	6
Female (n)	12	27	14
Age (years, mean ± SD)	55.4±12.7	55.3±12.2	45.2±12.9

*Additional healthy plasma were purchased from Innovative Research (https://www.innov-research.com/products/pooled-human-plasma-blood-derived): Pooled Human Plasma (Blood Derived).

Supplemental Table S2. Pathology of GBM patients, sex, and age

ID	Pathology	Sex	Age
J5-1	Glioblastoma: WHO grade 4<br>IDH wildtype<br>negative for MGMT promoter methylation<br>positive for point mutation in TERT promoter region<br>positive for EGFR amplification<br>negative for loss of PTEN	Female	67
J6-7	Diffuse astrocytoma: WHO grade 2<br>IDH1 mutant by IHC<br>negative for 1p36 deletion<br>negative for 19q13 deletion<br>polysomy and agin of 19q12 observed<br>negative for EGFR amplification<br>negative for loss of PTEN	Male	32
J8-1	Glioblastoma: WHO grade 4, with high cell density<br>IDH wildtype<br>negative for IDH1 R132H by IHC<br>positive for high level EGFR amplification<br>negative for whole chromosome 7 gain<br>positive for loss of PTEN, consistent with whole chromosome 10 loss<br>positive for point mutation in TERT promoter region (c.1-124C>T)<br>positive for MGMT promoter methylation	Female	65

J9-6	Glioblastoma: WHO grade 4<br>IDH-wildtype<br>negative for IDH1 R132H by IHC<br>positive for high level EGFR amplification<br>negative for whole chromosome 7 gain<br>positive for loss of PTEN, consistent with whole chromosome 10 loss<br>positive for MGMT promoter methylation<br>positive for point mutation in TERT promoter region (c.1-146C >T)<br>negative for mutation in IDH1, IDH2, and BRAF<br>positive for mutation in EGFR, anticipated single amino acid substitution (p.A289V)	Female	56
J5-5	Glioblastoma: WHO grade 4, clinically recurrent<br>IDH-wildtype<br>positive for EGFR amplification<br>positive for loss of PTEN<br>indeterminate for MGMT	Male	79
J10-3	Glioblastoma: WHO grade 4, with sarcomatous areas (gliosarcoma)<br>IDH-wildtype negative for IDH1 R132H by IHC<br>positive for high level EGFR amplification<br>negative for whole chromosome 7 gain<br>positive for loss of PTEN, consistent with whole chromosome 10 loss<br>negative for mutation in BRAF<br>negative for mutation in IDH1 and IDH2<br>negative for mutation in H3F3A<br>positive for point mutation in TERT promoter region (c.1-124C>T)<br>negative for deletion of CDKN2A (p16) sequences<br>positive for the presence of a KIAA1549-BRAF fusion product<br>positive for MGMT promoter methylation	Male	62

I21-6	Glioblastoma: WHO grade 4<br>IDH-wildtype negative for IDH1 R132H by IHC<br>rare cells with amplification of EGFR sequences<br>trisomy for 7p/7cen sequences, consistent with whole chromosome 7 gain<br>positive for loss of PTEN, consistent with whole chromosome 10 loss<br>positive for MGMT promoter methylation<br>positive for point mutation in TERT promoter region	Female	58
I24-6	Glioblastoma: WHO grade 4, with extensive necrosis<br>IDH-wildtype<br>history of clinically residual glioblastoma, IDH-wildtype, WHO grade 4<br>previous sample was positive for amplification of EGFR sequences<br>positive for loss of PTEN, consistent with monosomy 10	Female	64
I17-6	Glioblastoma: WHO grade 4, with primitive neuronal component<br>IDH mutant; positive for IDH1 R132H by IHC<br>negative for deletion of 1p36 sequences<br>positive for deletion of 19q13 sequences<br>negative for amplification of EGFR<br>trisomy and tetrasomy for 7p/7cen sequences, consistent with whole chromosome gain of 7<br>negative for loss of PTEN sequences and whole chromosome loss of 10;<br>trisomy and tetrasomy for PTE, consistent with whole chromosome gain of 10<br>positive for amplification of MYCN sequences<br>positive for amplification of CMYC sequences<br>MGMT promoter methylation not detected<br>TERT promotor mutation not detected	Female	28

I23-2	Glioblastoma: WHO grade 4, possibly arising in a ganglioglioma<br>IDH-wildtype negative for IDH1 R132H by IHC<br>negative for amplification of EGFR<br>trisomy for 7p/7cen sequences, consistent with gain of whole chromosome 7<br>positive for loss of PTEN consistent with whole chromosome 10 loss<br>negative for MGMT promoter methylation<br>negative for BRAF mutation<br>positive for point mutation in TERT promoter region (c.1-124C>T)<br>positive for EGFR mutation (p.T263P)	Male	43
J12-4	Glioblastoma: WHO grade IV; history of a left temporal anaplastic astrocytoma<br>IDH mutant<br>MGMT methylation indeterminate<br>very rare cells with EGFR amplification	Female	56
J12-2	Glioblastoma: WHO grade 4<br>IDH-wildtype<br>negative for IDH1 R132H by IHC<br>negative for amplification of MYCN<br>positive for trisomy and tetrasomy for chromosome 2<br>negative for amplification of EGFR<br>positive for multiple copies of 7p/7cen, consistent with whole chromosome 7 gain<br>borderline for loss of PTEN and 10 centromere (monosomy 10)<br>negative for MGMT promoter methylation<br>positive for point mutation in TERT promoter region	Male	60

J6-3	Glioblastoma: WHO grade 4 (clinically recurrent)<br>IDH-wildtype<br>negative for IDH1 IHC<br>negative for amplification of EGFR<br>positive for monosomy 10<br>indeterminate MGMT methylation<br>positive for TERT promotor mutations	Male	42
J3-6	Glioblastoma: WHO grade 4; clinically recurrent<br>history of right parietal glioblastoma, IDH-wildtype, WHO grade 4<br>positive for MGMT methylation<br>high level amplification of EGFR sequences<br>loss of PTEN, consistent with monosomy 10	Male	58
J3-1	Glioblastoma: WHO grade 4<br>IDH-wildtype<br>negative for IDH1 R132H by IHC<br>negative for amplification of EGFR<br>polysomy for 7p/7cen sequences, consistent with whole chromosome 7 gain<br>negative for deletion of 19q13 sequences<br>negative for loss of PTEN sequences or whole chromosome 10 loss<br>negative for deletion of 1p36 sequences<br>negative for MGMT promoter methylation<br>positive for point mutation in TERT promoter region (c.1-124C>T)	Male	80
J1-6	Glioblastoma: WHO grade 4 +<br>IDH-wildtype<br>positive for EGFR amplification<br>negative for whole chromosome 7 gain<br>positive for monosomy 10	Male	58
I24-4	Glioblastoma: WHO grade 4 (clinically recurrent)<br>IDH wildtype	Male	56

I24-3	Glioblastoma: WHO grade 4<br>IDH-wildtype<br>negative for IDH1/2 mutation<br>negative for IDH1 R132H by IHC<br>positive for mutation in H3F3A (p.G35R)<br>negative for H3K27M by IHC<br>strong diffuse p53 immunostaining<br>loss of nuclear ATRX<br>positive for amplification of EGFR sequences<br>polysomy for 7p12 (EGFR) and 7cen sequences, consistent with whole chromosome 7 gain<br>borderline for loss of PTEN and 10 centromere sequences<br>negative for amplification of MYCN (2p24.1) sequences<br>loss of MYCN (2p24.1) sequences<br>negative for amplification of C-MYC sequences<br>polysomy for 8q24 (C-MYC) and 8cen sequences<br>negative for MGMT promoter methylation<br>negative for point mutation in evaluated TERT promoter region	Male	33
I21-1	Glioblastoma: WHO grade 4<br>IDH-wildtype<br>negative for 1p/19q deletion<br>borderline for loss of 1p36 sequences<br>negative for deletion of 19q13 sequences<br>negative for amplification of EGFR<br>trisomy and tetrasomy for 7p/7cen sequences, consistent with gain of whole chromosome 7<br>positive for two populations with loss of PTEN sequences	Male	61

I20-2	Glioblastoma: WHO grade 4, epithelioid<br>IDH-wildtype<br>negative for IDH1 R132H by IHC<br>positive for amplification of EGFR sequences<br>no evidence for gain of whole chromosome 7<br>positive for loss of PTEN, consistent with loss of whole chromosome 10<br>positive for point mutation in promoter region of TERT<br>no BRAF mutation detected	Male	59
I18-1	Glioblastoma: WHO grade 4, clinically recurrent<br>IDH-wildtype<br>negative for IDH1 R132H by IHC<br>positive for amplification of EGFR sequences<br>negative for whole chromosome 7 gain<br>positive for loss of PTEN, consistent with a loss of whole chromosome 10	Male	56
J3-3	Glioblastoma: WHO grade 4, recurrent; on clinical trial<br>IDH wildtype	Male	56
I17-2	Glioblastoma: WHO grade 4<br>IDH-wildtype<br>negative for IDH1 R132H by IHC<br>positive for amplification of EGFR sequences; no evidence of whole chromosome 7 gain<br>positive for loss of PTEN, consistent with whole chromosome loss of 10<br>postivie for TERT promoter variant, c.1-124C>T<br>MGMT promoter methylation not detected	Male	46
E17-5	GBM, according to Pathology	Male	57
E19-6	Glioblastoma: WHO Grade IV, with oligodendroglial component, and acute hemorrhage<br>IDH1 positive by IHC<br>negative for 1;36 deletion<br>negative for 19q13 deletion<br>negative for EGFR amplification<br>positive for loss of PTEN, consistent with monosomy 10	Female	32

E11-5	Glioblastoma: WHO grade 4<br>negative for MGMT promoter methylation	Male	64
E17-2	GBM, according to Pathology	Female	49
E17-3	GBM, according to Pathology	Male	70
E19-6	GBM, according to Pathology	Female	32
E12-2	Glioblastoma: WHO grade 4<br>IDH wildtype<br>IDH-1 negative by IHC<br>positive for high level of EGFR amplificaiton<br>loss of PTEN, consistent with monosomy 10<br>negative for MGMT methylation	Male	60
I18-4	Glioblastoma: Grade 4<br>point mutation in TERT promoter region	Male	64
I17-7	Glioblastoma: WHO grade 4<br>IDH-wildtype<br>negative for IDH1 R132H by IHC<br>positive for amplification of EGFR sequences<br>negative for whole chromosome 7 gain<br>positive for loss of PTEN, consistent with whole chromosome loss of 10<br>TERT Promoter variant c.1-124C>T<br>MGMT promoter methylation detected	Male	65
I17-5	Glioblastoma: WHO grade 4 (clinically recurrent)<br>IDH-wildtype<br>prior resection specimen negative for MGMT methylation<br>positive for TERT promoter mutation<br>positive for EGFR amplification	Female	58
E17-4	GBM, according to Pathology	Female	66
E13-7	Glioblastoma: WHO grade 4, with epithelioid features<br>negative for MGMT methylation<br>negative for EGFR amplification<br>borderline positive for PTEN loss, consistent with monosomy 10	Male	36

E13-1	Glioblastoma: WHO grade 4<br>IDH wildtype<br>IDH1 negative by IHC<br>IDH1 negative for mutation<br>strong nuclear p53 IHC<br>strong EGFR expression<br>positive for amplification of EGFR sequences<br>negative for loss of PTEN sequences<br>MGMT methylation detected	Male	56
E11-1	Glioblastoma: WHO grade 4<br>IDH wildtype<br>IDH1 negative by IHC<br>positive for EGFR amplification<br>negative for PTEN loss<br>MGMT methylation not detected	Male	74
J13-6	Glioblastoma: WHO grade 4, clinically recurrent right temporal glioblastoma<br>IDH-wildtype<br>negative for IDH1 R132H by IHC<br>positive for amplification of EGFR sequences<br>positive for loss of PTEN, consistent with monosomy 10<br>MGMT promoter methylation not detected<br>no fusion or potentially oncogenic transcripts identified in ALK, ROS1 or MET<br>EGFR-SEPT14 fusion transcript identified [EGFR exon 24 to SEPT14 exon 10]<br>TERT promoter variant identified (c.1-124C>T).	Male	51
J13-3	Glioblastoma: WHO grade 4<br>positive for EGFR amplification<br>positive for loss of PTEN consistent with whole chromosome 10 loss	Male	56

E20-7	Anaplastic astrocytoma: WHO grade 3, clinically gliomatosis cerebri by history<br>IDH wildtype<br>IDH1 IHC negative<br>IDH1, exon 4: no mutation detected<br>IDH2, exon 4: no mutation detected<br>EGFR protein expression negative<br>ATRX retained	Male	50

Supplemental Table S3. MMA patient ID, pathology, sex and age

ID	Pathology	Sex	Age
J11-2	Meningioma: WHO grade 1	Female	58
J11-7	Meningioma: WHO grade 1	Male	59
J10-7	Meningioma: WHO grade 1	Male	48
J10-6	Meningioma: WHO grade 1; point mutation in TERT promoter region	Male	55
J8-6	Meningioma: WHO grade 1	Female	43
J9-3	Meningioma: WHO grade 1	Female	70
J4-7	Meningioma: WHO grade 1	Female	36
J7-5	Meningioma: WHO grade 1	Female	46
J7-6	Meningioma: WHO grade 1	Female	58
J6-2	Meningioma: WHO grade 1	Female	57
E6-4	Meningoma; recurrent	Female	75
E6-1	Meningioma: WHO grade 1	Female	36
E8-3	Meningioma: WHO grade 1; angiomatous variant with focal atypical features	Male	63
E5-3	Meningioma: WHO grade 1	Female	64
I22-6	Meningioma: WHO grade 1	Female	48
I19-2	Meningioma: WHO grade 1	Female	53
J4-7	Meningioma: WHO grade 1	Female	36
J4-1	Meningioma: WHO grade 1	Female	64
I20-7	Meningioma: WHO grade 1	Male	74
J1-4	Meningioma: WHO grade 1; extensive transdural and dural sinus involvement	Female	39
E4-6	Meningioma: WHO grade 1	Female	62
E8-1	Meningioma: WHO grade 1	Female	53
E8-2	Meningioma: WHO grade 2 (atypical)	Male	41

J3-2	Meningioma: WHO grade 1; clinically radiation-induced	Male	74
J2-2	Meningioma: WHO grade 1	Female	65
I18-5	Meningioma: WHO grade 1	Female	37
J4-4	Meningioma: WHO grade 1	Male	67
J11-6	Meningioma: WHO grade 1	Female	57
I15-5	Meningioma: WHO grade 1	Female	43
I20-4	Meningioma: WHO grade 1	Male	66
E4-5	Meningioma: WHO grade 1	Male	74
E15-6	Meningioma: WHO grade 1	Female	43
E6-2	Meningioma: WHO grade 1; psammomatous type	Male	37
I18-2	Meningioma: WHO grade 1	Female	55
I24-2	Meningioma, WHO grade I	Female	75
J5-7	Meningioma: WHO grade 1; no TERT mutation found	Female	51
J4-2	Meningioma: WHO grade 1	Female	49
J10-2	Meningioma: WHO grade 1	Male	64
I19-3	Meningioma: WHO grade 1	Female	53
I21-4	Meningioma: WHO grade 1; psammomatous subtype	Female	63

Supplemental Table S4. Healthy Control ID, sex and age

Healthy Control ID	Sex	Age
ACP-HC1	F	51
ACP-HC2	F	54
ACP-HC3	F	29
ACP-HC4	F	43
ACP-HC5	F	24
ACP-HC6	F	23
ACP-HC7	F	44
ACP-HC8	F	61
ACP-HC9	F	30
ACP-HC10	F	57
ACP-HC11	F	55
ACP-HC12	F	54
ACP-HC13	F	49
ACP-HC14	F	43
ACP-HC15	M	61
ACP-HC16	M	57
ACP-HC17	M	40

Supplemental Table S5. Phage peptides identified by brain tumor EVs

EV ID	Phage ID	Peptide	EV ID	Phage ID	Peptide
GBM pooled plasma EVs	JC1a/C2	SKADWNMAEAYF	MMA pooled plasma EVs	JC1a/D2	FETTVLMDLYRY
	JC1a/C3	NHAHYLGALLST		JC1a/D11	YLDAHFYSEGAK
	JC1a/C5	MLPIKQEYHFPI		JC1a/D7	SNTWTWISSAYG
	JC1a/C6	TILKPAAQGFAD		JC1a/D9	FAKPDMRSNLGW
	JC1a/C7	AEAXTGFSASGV		JC1a/D10	VNASDFDFSRKK
	JC1a/C8	AEAWTGFSASGV	MMA plasma EVs	JC1a/E2	SIVKTHTQIDIF
	JC1a/C9	AEAWTGFSASGV		JC1a/E3	YSPVLIW
	JC1a/C12	AEAWTGFSASGV		JC1a/E4	QQMHEWYFQSLP
GBM plasma EVs	JC1a/F1	GWNEAVNLMDSM		JC1a/E6	DHAMHQNQNISN
	JC1a/F3	SHYQGNHVDNKL		JC1a/F7	SHWGSDHQSVMT
	JC1a/F4	LMPVTPK		JC1a/F12	SHYQGNHVDNKL
	JC1a/F5	NHAHYLGALLST		JC1a/F9	QMYGLNIGGSWT
	JC1a/F6	TMVLAYDKTADI		JC1a/F10	NHAHYLGALLST
	JC1a/E9	TILKPAAQGFAD		JC1a/F11	DVSAILYRSGFP
	JC1a/E7	TILKPAAQGFAD	MMA cell line EVs	E9-2MF1.G10	VHWDFRQWWQPS
	JC1a/E8	NDIITSEQFKQG		E9-2MF2.C8	VHWDFRQWWQPS
	JC1a/E10	ISYDRGSFEAQS		E9-2MF1.A9	VHWDFRQWWQPS
	JC1a/E11	WAGLVSWMPNFS		E9-2MF2.B9	IGRMVTLEPGVM
	JC1a/E12	TPSSDNYLRTHN		E9-2MF2.B10	FEVNGTDHLPIH
GBM cell line EVs	F3-8 JC1a	HQMMLQAQPVKN		E9-2MF2.C9	IRNHVETINAGI
	F3-8 SF1	HLYALMT		E9-2MF2.H9	SFDDNVGQPPSP
	F3-8 JC1a	WIPRFTD		E9-2MF2.H10	HFVPLVNLGVLS

Supplemental Table S6. For EVs derived from similar sources, EV-binding peptides reveal conserved sequences (https://www.ebi.ac.uk/Tools/msa/clustalo/)

GBM pooled plasma EVs	
JC1a/C7	AEAXTGFSASGV
JC1a/C8	AEAWTGFSASGV
JC1a/C9	AEAWTGFSASGV
JC1a/C12	AEAWTGFSASGV
	*** ********
GBM plasma EVs	
JC1a/F6	-TMV---LAYDKTADI
JC1a/E9	-TILK--PAAQGFAD-
JC1a/E7	-TILK--PAAQGFAD-
JC1a/E8	NDIITSEQFKQG----
	:: :
MMA pooled plasma EVs	
JC1a/D9	FAKPDMRSNLGW------
JC1a/D7	------SNTWTWISSAYG
JC1a/D2	FETTVLMDLYRY------
JC1a/D11	-----YLDAHFYSEGAK-
JC1a/D10	------VNASDFDFSRKK
	. :
MMA plasma EVs	
JC1a/E6	DHAMHQNQNISN--
JC1a/F7	--SHWGSDHQSVMT
JC1a/F12	--SHYQGNHVDNKL
JC1a/F9	--QMYGLNIGGSWT
	: .

Supplemental Table S7. BLAST searches of peptide sequences showing homologies to known proteins

Peptide ID	Peptide sequence	Protein name	Identity	Protein name	Identity
GBM cell line XO-1	HLYALMT	focal adhesion kinase 1 isoform X1	5/6(83%)	phosphatidylinositol 4,5-bisphosphate 3-kinase catalytic subunit delta isoform isoform X1	5/6(83%)
GBM cell line XO-2	HTWLHAVQPRFA	immunoglobulin heavy chain junction region	7/9(78%)	autism susceptibility gene 2 protein isoform X1	6/9(67%)
GBM cell line XO-4	HQMMLQAQPVKN	atrophin-1 related protein	7/9(78%)	arginine-glutamic acid dipeptide (RE) repeats, isoform CRA_b	7/9(78%)
GBM cell line XO-5	WIPRFTD	immunoglobulin heavy chain variable region, partial	5/5(100%)	deleted in malignant brain tumors 1 protein isoform e precursor	6/7(86%)
GBM Pl-2	VHWDFRQWWQPS	immunoglobulin heavy chain junction region	5/7(71%)	anti-HIV-1 immunoglobulin heavy chain variable region, partial	5/6(83%)
GBM PXO	GPRPSDLTMATR	serine/threonine-protein phosphatase 4 regulatory subunit 1 isoform X1	9/14(64%)	immunoglobulin heavy chain junction region	7/9(78%)
GBM PPXO	SKADWNMAEAYF	immunoglobulin heavy chain junction region	7/8(88%)	sodium/hydrogen exchanger 10 isoform 1	6/7(86%)
GBM PPXO JC1a/C3	NHAHYLGALLST	probable E3 ubiquitin-protein ligase HERC1 isoform X1	7/8(88%)	teneurin-4 isoform X1	6/6(100%)
GBM PPXO JC1a/C5	MLPIKQEYHFPI	leucine-rich repeat transmembrane protein FLRT1	10/20(50%)	immunoglobulin light chain junction region	5/7(71%)
GBM PPXO JC1a/C6	TILKPAAQGFAD	TPA_inf: olfactory receptor OR17-8	8/10(80%)	immunoglobulin heavy chain junction region	7/8(88%)

GBM PPXO JC1a/C7	AEAXTGFSASGV	immunoglobulin heavy chain junction region [Homo sapiens]	7/10(70%)	ATPase WRNIP1 isoform 1 [Homo sapiens]	8/11(73%)
GBM PXO	SIVKTHTQIDIF	MHC class I antigen, partial	7/8(88%)	oxysterol-binding protein-related protein 10 isoform X4	8/11(73%)
GBM PXO JC1a/E10	ISYDRGSFEAQS	immunoglobulin heavy chain junction region	7/9(78%)	GTP-binding protein REM 1 isoform X1	7/9(78%)
GBM PXO JC1a/E11	WAGLVSWMPNFS	arachidonate 12-lipoxygenase, 12R-type	5/5(100%)	immunoglobulin heavy chain junction region	5/6(83%)
GBM PXO JC1a/E12	TPSSDNYLRTHN	immunoglobulin heavy chain junction region	7/9(78%)	dmX-like protein 1 isoform X1	6/6(100%)
GBM PXO JC1a/E3	YSPVLIW	protein transport protein Sec16A isoform X1	6/6(100%)	immunoglobulin heavy chain junction region	6/7(86%)
GBM PXO JC1a/E4	QQMHEWYFQSLP	mitogen-activated protein kinase kinase kinase 15	6/7(86%)	immunoglobulin heavy chain junction region	5/6(83%)
GBM PXO JC1a/E6	DHAMHQNQNISN	alternative protein DIRAS2 [Homo sapiens]	6/11(55%)	chromodomain-helicase-DNA-binding protein 9 isoform X1	5/6(83%)
GBM PXO JC1a/E8	NDIITSEQFKQG	PDS5, regulator of cohesion maintenance, homolog A	6/8(75%)	BAH and coiled-coil domain-containing protein 1 isoform 1	6/6(100%)
MMA cell line XO-2	SFDDNVGQPPSP	aprataxin and PNK-like factor	6/7(86%)	Structure of CHD4 double chromodomains depicts cooperative folding for DNA binding	6/6(100%)
MMA cell line XO-3	FEVNGTDHLPIH	serine/threonine-protein phosphatase 6 regulatory ankyrin repeat subunit C	6/7(86%)	immunoglobulin heavy chain junction region	6/8(75%)
MMA cell line XO-4	IRNHVETINAGI	very-long-chain (3R)-3-hydroxyacyl-CoA dehydratase 3	7/8(88%)	dynein, axonemal, heavy polypeptide 7, isoform CRA_b	8/11(73%)

MMA cell line XO-5	HFVPLVNLGVLS	tumor necrosis factor alpha-induced protein 3	7/8(88%)	Peripherin 2 (retinal degeneration, slow)	9/14(64%)
MMA cell line XO-6	IGRMVTLEPGVM	immunoglobulin heavy chain junction region	7/8(88%)	zinc finger protein 469	7/10(70%)
MMA PPXO	FETTVLMDLYRY	methylcytosine dioxygenase TET2 isoform a	6/6(100%)	fetal liver non-specific cross-reactive antigen-3 precursor protein [Homo sapiens]	6/7(86%)
MMA PPXO JC1a/D10	VNASDFDFSRKK	immunoglobulin heavy chain junction region	7/8(88%)	inaD-like protein isoform X1	6/7(86%)
MMA PPXO JC1a/D11	YLDAHFYSEGAK	immunoglobulin heavy chain junction region	6/6(100%)	autophagy-related protein 2 homolog A isoform X1	7/9(78%)
MMA PPXO JC1a/D7	SNTWTWISSAYG	immunoglobulin heavy chain variable region, partial	7/9(78%)	IG c804_heavy_IGHV4-39_IGHD6-13_IGHJ4, partial	6/7(86%)
MMA PPXO JC1a/D9	FAKPDMRSNLGW	protein AHNAK2 isoform 1 [Homo sapiens]	7/8(88%)	immunoglobulin light chain junction region [Homo sapiens]	6/6(100%)
MMA PXO	GWNEAVNLMDSM	histone H2B type F-M isoform X1 [Homo sapiens]	7/9(78%)	PRKC, apoptosis, WT1, regulator, isoform CRA_a	7/8(88%)
MMA PXO JC1a/F11	DVSAILYRSGFP	Rh-associated glycoprotein [Homo sapiens]	7/9(78%)	50 kDa erythrocyte plasma membrane glycoprotein [Homo sapiens]	7/9(78%)
MMA PXO JC1a/F3	SHYQGNHVDNKL	immunoglobulin heavy chain junction region	8/11(73%)	cytochrome P450 27C1 isoform 2	6/6(100%)
MMA PXO JC1a/F4	LMPVTPK	serine/threonine-protein kinase D3	6/6(100%)	PRKD3 protein	6/6(100%)
MMA PXO JC1a/F6	TMVLAYDKTADI	E3 ubiquitin-protein ligase MARCHF9 precursor	8/10(80%)	membrane-associated ring finger (C3HC4) 9, isoform CRA_b	8/10(80%)
MMA PXO JC1a/F9	QMYGLNIGGSWT	immunoglobulin heavy chain junction region	7/10(70%)	armadillo repeat containing, X-linked 4, isoform CRA_a	6/6(100%)

Notes:

ppXO: Pooled plasma EVs

pXO: Individual plasma EVs

Pl: Plasma

Supplemental Table S8. List of synthetic peptides with high affinity to corresponding EVs

Pep ID	Sequence (N--C)	Molecular Weight	Water Solubility	Notes
GBM PPXO C2	SKADWNMAEAYF	1772	Poor	EVs from GBM pooled plasma
GBM PPXO E5	GPRPSDLTMATR	1641	Poor	EVs from GBM pooled plasma
MMA-D2	FETTVLMDLYRY	1550	Poor	EVs from meningioma pooled plasma
MMA JC1A/D11	YLDAHFYSEGAK	1440	Good	EVs from patient plasma
F3-8 JC1A/B4	WIPRFTD	934	Good	Surface pan. EVs from GBM cell line
F3-8 (SF1)F5	HLYALMT	1073	Poor	Surface pan. EVs from GBM cell line
HC-XO G1	LVVPHRSLSEVR	1391	Good	EVs from commercial HC plasma
HC-XO G4	FSGLSLTRMFYK	1449	Poor	EVs from commercial HC plasma
